# Skin Immunity and Tolerance: Focus on Epidermal Keratinocytes Expressing HLA-G

**DOI:** 10.3389/fimmu.2021.772516

**Published:** 2021-12-06

**Authors:** Guillaume Mestrallet, Nathalie Rouas-Freiss, Joel LeMaoult, Nicolas O. Fortunel, Michele T. Martin

**Affiliations:** ^1^ Commissariat ã l’Energie Atomique et aux Energies Alternatives, DRF, Francois Jacob Institute of Biology, Laboratory of Genomics and Radiobiology of Keratinopoiesis, Institute of Cellular and Molecular Radiobiology, Evry, France; ^2^ Université Paris-Saclay, Saint-Aubin, France; ^3^ Commissariat ã l’Energie Atomique et aux Energies Alternatives, DRF, Francois Jacob Institute of Biology, Hemato-Immunology Research Department, Saint-Louis Hospital, Paris, France; ^4^ Université de Paris, UMR-S 976 HIPI Unit, Paris, France

**Keywords:** immunity, tolerance, human skin, keratinocytes, resident immune cells, HLA-G

## Abstract

Although the role of epidermal cells in skin regeneration has been extensively documented, their functions in immunity and tolerance mechanisms are largely underestimated. The aim of the present review was to outline the state of knowledge on resident immune cells of hematopoietic origin hosted in the epidermis, and then to focus on the involvement of keratinocytes in the complex skin immune networks acting in homeostasis and regeneration conditions. Based on this knowledge, the mechanisms of immune tolerance are reviewed. In particular, strategies based on immunosuppression mediated by HLA-G are highlighted, as recent advances in this field open up perspectives in epidermis-substitute bioengineering for temporary and permanent skin replacement strategies.

## Skin Cells Ensure Tissue Protection and Homeostasis

The skin accounts for 15% of body weight, provides an exchange surface between organism and environment, and protects internal organs. It also helps to maintain homeostasis by preventing water loss and by regulating body temperature. The epidermis, the outermost layer of the skin, is composed of keratinocytes (90% of cells). It also contains melanocytes (5%) and rare Merkel cells. This barrier protects the underlying skin layers from injury, UV damage, harmful chemicals and infection by pathogens. The dermis, separated from the epidermis by the dermo-epidermal junction, is composed of extracellular matrix secreted by fibroblasts. It contains blood vessels, glands and nerve cells. Its main functions are to deliver oxygen and nutrients to the epidermis and to regulate body temperature.

**Figure 1:**
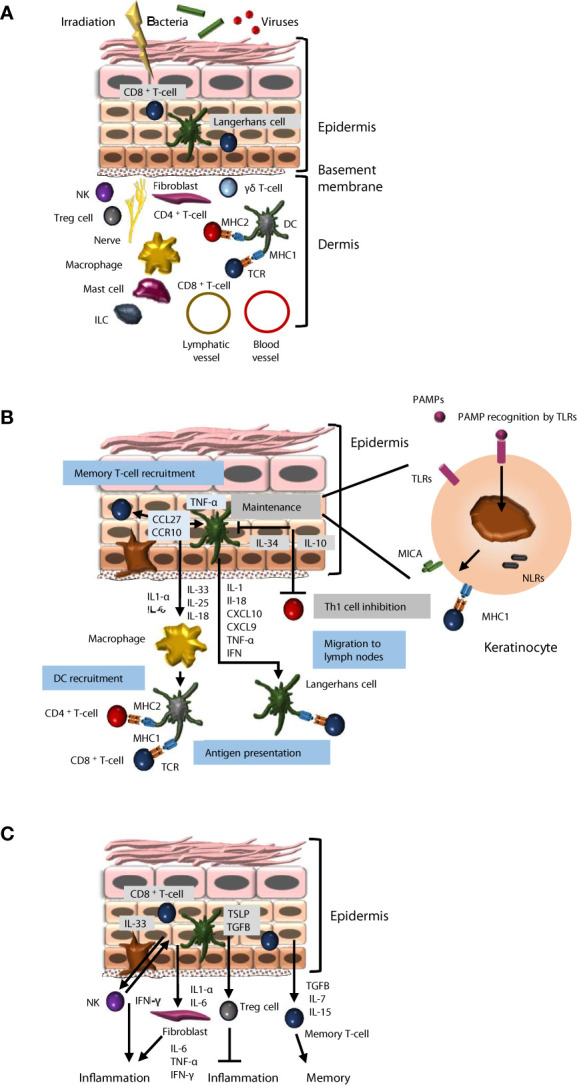
Immune properties of skin keratinocytes. **(A)** Cellular components of the skin immune system. The skin is a barrier that protects against injury, pathogens, chemicals and radiation. The local immune system includes resident immune cells and cells recruited from the periphery. The epidermis hosts effector cells, such as CD8^+^ T-cells and Langerhans cells that migrate to lymph nodes to perform antigen presentation. In the dermis, Treg cells, NK, CD4^+^ T-cells, mast cells, ILCs, macrophages and dendritic cells participate in immune activities. In addition, resident non-hematopoietic skin cells have immune functions, which are not fully elucidated. **(B)** Keratinocyte interactions with immune cells. Keratinocytes recognize pathogen-associated molecular patterns (PAMPSs) through TLR and NLR receptors and stimulate the recruitment of resident memory T-cells, dendritic cells and circulating T-cells through pro-inflammatory cytokine secretion. On the other hand, they are also able to promote tissue homeostasis through anti-inflammatory cytokine secretion (IL-34, IL-10). **(C)** Keratinocytes participate in the regulation of inflammation and the maintenance of skin immune memory. Keratinocytes promote inflammation through IL-1, IL-3 and IL-6 secretion, but also limit it through TSLP and TGFB secretion. Keratinocytes can promote the maintenance of immune memory through various mechanisms, including TGFB, IL-7 and IL-15 secretion.

Adult skin contains resident immune cells and recruits immune cells from the periphery in case of infection, burns or exposure to chemicals or radiation. Resident immune cells are found in all layers of the tissue, which therefore constitutes a reservoir of immune cells (1) (**Figure 1A**), and notably of T-cells. It was estimated that adult skin contains 20 billion T-cells, nearly twice as many as in the blood (2). In addition, resident non-hematopoietic skin cells have immune functions, which are not fully elucidated.

## The Epidermis Is the Seat of Immune Activities

### Immune Cells Reside in the Epidermis

As a direct interface with the environment, the epidermis hosts various immune cells, and notably tissue-resident memory αβ CD8^+^ memory T-cells, dendritic epidermal gamma delta T-lymphocytes (γδ T-cells), and Langerhans cells (LCs, 5% of epidermal cells). LCs originate prenatally from erythromyeloid progenitors and permit antigen presentation to T-cells (3). They are in contact with keratinocytes *via* their dendrites and with dendritic cells below the dermo-epidermal junction. LCs produce inflammatory mediators such as interferons (IFNs), and enable presentation of antigens to other immune actors such as T-cells (4). LCs express CD1a (5), CD207 and MHC class II (MHC2), and have cytoplasmic Birbeck granules. In immune reactions, they express MMP proteases, translocate to the dermis, and migrate to lymph nodes, promoting T-cell recruitment (1), activation of CD8^+^ T-cells, and differentiation of type 2 T-helper lymphocytes (Th2) (6). In short, LCs exert a regulatory role in the lymphocyte activation cascade. In case of tissue destruction, LCs are renewed by differentiation of monocytes recruited at the lesion site (7). LCs are able to divide and self-maintain, like macrophages, and to migrate to lymph nodes and stimulate T-cells, like dendritic cells (DCs), thus sharing both macrophage and DC properties.

Within the skin reservoir of resident T-cells, the most frequent subtype is αβ CD8^+^ memory T-cells, localized in the basal and supra-basal layers next to LCs (8). These cells express cutaneous lymphocyte-associated antigen (CLA), a skin-homing receptor (2), and different chemokine receptors (CCR4, CCR8 and CCR10) (9). They also express the IL2 receptor (CD25) and HLA-DR (10). Tissue-resident innate lymphoid cells (ILCs) were recently described in epidermis, predominantly expressing ILC3/LTi-related genes or genes associated with ILC2s, but their role remains to be investigated (11).

### Molecular Effectors Related to Immune Functions Are Expressed by Keratinocytes

#### Membrane Markers

Pathogens are identified *via* pathogen-associated molecular patterns (PAMPs), recognized by pattern recognition receptors (PRRs) (12). Major PRRs are toll-like receptors (TLRs), expressed as 8 isoforms in keratinocytes (13) (**Figure 1B**). Keratinocytes also express nucleotide-binding oligomerization domain (NOD)-like receptors (NLRs). NLR signaling leads to activation of the inflammasome *via* NLRP3, an intracellular sensor that detects microbial motifs, and the production of pro-inflammatory cytokines such as IL-1β and IL-18. NOD1 and NOD2 are expressed by keratinocytes, and drive inflammatory signals *via* the NF-κB and MAPK pathways [8]. TLRs and NLRs promote detection of alarmins released from or exposed at the surface of damaged cells (9). PAMP binding to TLRs leads to the production of cytokines (IL-1β, IL-18, CXCL9 and CXCL10) and recruitment of immune cells.

TLRs recognize different ligands, enabling efficient pathogen detection. TLR1, 2, 6 and 10 recognize lipoproteins, while TLR3, 4 and 5 respectively recognize RNA, bacterial LPS, and flagellin. TLR7, 8 and 9 recognize RNA and DNA. Most TLRs have a pro-inflammatory function, except TLR10 (14). NF-κB signaling is the major pathway driving TLR-mediated cellular response, leading to the production of IFNs (15). This cascade triggers LC migration to lymph nodes, inducing recruitment of T-cells expressing the skin diapedesis marker CLA (16). IFN-γ up-modulates TLR3 expression in keratinocytes, which in turn increase their secretion of IL-6, Il-8 and defensins, in the presence of the immune stimulant molecule polyinosinic:polycytidylic acid (poly I:C) (17). Poly I:C binding to TLR3 stimulates keratinocyte production of IL-1β and IL-18, promoting DC activation and T-cell differentiation into Th1 (9). The complex TLR3 signaling network in keratinocytes involves production of chemokines CXCL9 and CXCL10 and of cytokines CCL20 and CCL27, which respectively promote the recruitment of Th1 and memory T-cells (9).

Keratinocytes express MHC components, allowing antigen recognition, and notably MHC1-related cell-surface molecules (MICA, MICB and ULBP) that mediate interactions with CD8+ T and γδ T-cells through the natural-killer group 2D receptor (NKG2D). NKG2D is an immune reaction promoter expressed by resident or infiltrating immune cells in the skin (18). A key functional link is that ligand binding to TLRs induces expression of some HLA molecules (13). For example, poly I:C, a TLR3 ligand, induces expression of HLA-ABC and HLA-DR. Similarly, flagellin, a TLR5 ligand, induces expression of HLA-ABC, and LPS, a ligand of TLR4, induces expression of HLA-DR. MHC components are not present in all keratinocytes, as a basal sub-population expresses neither MHC1 nor MHC2 (19).

#### Secreted Factors

Keratinocytes secrete antimicrobial peptides (AMPs), damage-associated molecular patterns (DAMPs) and the defensin peptides that ensure direct antimicrobial functions. Keratinocytes secrete antimicrobial proteins (S100) and peptides (cathelicidins) (9). AMP secretion by keratinocytes during infection increases following production of IL-17A and IL-22 by Th17-cells (20) and in response to IFN-γ (21), which amplifies the inflammatory response. Secreted mediators include pro- or anti-inflammatory cytokines, CXC and CC chemokines and growth factors (22). These mediators constitute the signaling network linking epidermal and dermal cells to resident immune cells to maintain skin homeostasis and defense against environment insults.

### Keratinocytes and Immune Cells Communicate Directly

#### *Via* Cell-Cell Contact

Through MHC1 molecules, keratinocytes present antigens to memory CD8+ T-cells, inducing cytotoxic defenses and production of inflammatory cytokines (23), notably following stimulation by IFN-γ. Antigen presentation *via* MHC2 molecules was documented in a mouse skin model where interfollicular keratinocyte MHC2 drives the formation of Th1-cell clusters (24). Keratinocytes express adhesion molecules such as ICAM-1, which, with its B7 costimulatory molecules, promotes lymphocyte recruitment (25).

Keratinocytes exert a role in the coordination of differentiation and functions of effector T-cells, natural-killer T (NKT) cells (26) and γδ T-cells (27). Target cell detection by NKT and γδ T-cells involves the NKG2D receptor, which recognizes the MICA and MICB proteins. NKG2D-mediated signaling leads to target-cell lysis (28). Overexpression of MICA and MICB in damaged keratinocytes drives a recognition signal by NKT and γδ T-cells, and their lysis (9). A major role of NKT cells is protection against microbial infection, through recognition of bacterial glycolipids (26).

#### *Via* Secretion of Cytokines and Chemokines

Primary cytokines (IL-1, TNF-α) are secreted by keratinocytes in the initial stage of inflammatory reactions. Interleukin-1 exists in 3 isoforms (IL-1α, IL-1β and IL-1Ra), which bind to the same receptors (IL-1R1 and IL-1R2). Keratinocytes produce and store active IL-1α (29), and produce an inactive pro-IL-1β form that is cleaved and activated by caspase-1 synthesized by LCs, mast cells, monocytes, macrophages and neutrophils. IL-1α promotes its own expression and that of other pro-inflammatory cytokines (IL-6, IL-8, TNF-α). It induces expression of adhesion molecules promoting tissue infiltration by immune cells. Dysregulation of IL-1 signaling in keratinocytes was associated with mutations in the *NLRP1* inflammasome sensor gene in patients with inflammatory skin syndrome (30). Secretion of IL-1β induced *via* the TLR4-MAPK pathway in keratinocytes promotes early skin-wound healing (31). Production of IL-1 and IL-18 by keratinocytes following exposure to UV promotes recruitment of Th1 and Th2 cells, which themselves produce interleukins, TNF-α and CSF2 (32). IL-18 is produced by keratinocytes in an inactive form (33). Following UV exposure, inflammasome activation is driven by the stress sensor NLRP3, leading to pro-IL-18 cleavage by caspase-1 (34). Moreover, keratinocyte production of IL-6 promotes proliferation and differentiation of B-cells and cytotoxic T-cells (35).

Transforming growth factor beta (TGFB) is produced by epidermal keratinocytes, and plays an important role in skin remodeling after damage (36). TGFB inhibits macrophage differentiation, monocyte and CD8^+^ T-cell activity, and presentation of antigens by LCs (37). In keratinocytes, TGFB inhibits proliferation of CD4+ T-cells through Smad3 signaling (38). In inflammatory contexts, TGFB promotes leukocyte adhesion and chemotaxis, and activates DC migration into lymph nodes, promoting T-cell recruitment in the skin (36).

IL-33 may either increase or inhibit inflammation. The inactive form, pro-IL-33, is located in the cell nucleus, where it suppresses transcription of pro-inflammatory cytokines (39). In keratinocytes, nuclear IL-33 inhibits epidermis differentiation genes in atopic dermatitis lesions, exacerbating skin barrier dysfunction (40). The active form after cleavage allows TH2 cytokine signaling *via* the IL-1 receptor ST2. In keratinocytes, IL-33 secretion is induced by cytokines and by pathogens such as S aureus (41). IL-25, like IL-33 and IL-1α, is stored in keratinocytes and secreted under the action of proteases during damage, contributing to immune response activation (42). Keratinocytes also produce the anti-inflammatory cytokine IL-10 (43), which may reduce formation of large scars (44).

Chemokines are of great importance for immune cell recruitment and mobility within tissue (45). They contribute to regulation of immune cell activation and differentiation (46). Keratinocytes express chemokines of both the CC and CXC families. CXC chemokines attract neutrophils during healing, while CC chemokines attract a wider range of leukocytes: basophils, eosinophils, T-cells and DCs (47).

In tissue injury, cytokines and chemokines contribute to skin repair through interaction with keratinocyte stem cells. In mouse skin, resident Tregs activate hair-follicle stem cells (HFSCs) by secreting the CXCL5-IL-17- IFN-γ signal. In response, HFSCs are recruited and migrate to the interfollicular epidermis, and contribute to on-site epithelial-barrier repair (48). Interactions between keratinocytes and effector T-cells have reciprocal impacts: keratinocytes promote effector T-cell recruitment within the skin, and these in turn produce growth factors such as CTGF, FGF9, KGF and IGF1, that promote healing (49).

#### Other Communication Mechanisms

Keratinocytes communicate with other cells *via* secretion of extracellular vesicles containing cargoes of different types of molecule, including lipids, proteins and nucleic acids (50). Epigenetic mechanisms are also emerging, as illustrated by the study of the Mi-2β chromatin remodeler. In mouse skin, this factor directly controls regulatory T-cells by inhibiting pro-inflammatory TSLP secretion by keratinocytes (51).

### Keratinocytes Mediate Inflammation

Keratinocytes are involved in the initiation of inflammatory processes, through release of soluble mediators, including IFN-γ (**Figure 1C**). Keratinocytes are activated before ‘true’ immune cells at the onset of inflammation (25). IFN-γ has a central pro-inflammatory function in the skin, and a single intradermal injection of IFN-γ is sufficient to induce an inflammatory state, driven by a cytokine production cascade (52). Keratinocytes are the primary cellular actors in a positive loop, as, following exposure to IFN-γ, they increase their secretion of IL-33, which in turn increases their production of IFN-γ (53).

Studies in a mouse model showed that a subpopulation of keratinocytes expressing PD-L1 also promotes control of the extent of inflammation (54). Human keratinocytes promote local but not systemic inflammation, through expression of thymic stromal lymphopoietin (TSLP), a factor involved in Treg immune function coordination. By binding to its receptor on Tregs, TSLP maintains local inflammation while inhibiting lethal systemic inflammation (9). In addition, presentation of autoantigens by keratinocytes induces T-cell tolerance, and is a means of avoiding, rather than stimulating, autoimmune reactions in contexts of local inflammation (51).

### Keratinocytes Promote Immune Memory

The skin hosts resident memory T-cells that favor rapid response to infection by a pathogen to which the individual has already been exposed (55). A model of cutaneous immune response in three successive stages was proposed (**Figure 1C**) (56). First, following pathogen entry into tissue, resident specific memory T-cells resulting from previous exposure react by transcriptional changes and secretion of activating factors. Second, circulating memory T-cells are recruited. And third, skin DCs presenting antigens of the pathogen migrate to lymph nodes, where they drive neo-production of specifically targeted effector T-cells, which are recruited in the infection site in the skin, within 24 to 72 hours (57). Keratinocytes promote long-term maintenance of a memory T-cell pool within the skin, through secretion of IL-7, IL-15 and TGFB, favoring a rapid defense response in case of new aggression by a previously encountered pathogen (9). A memory mechanism that does not require skin-resident macrophages or T cells has been identified in murine epidermal stem cells after acute inflammation (58). Stem cells maintain prolonged epigenetic memory to acute inflammation by maintaining chromosomal accessibility to stress response genes, which, in case of secondary stress, enables fast transcription of specific inflammasome genes, including the *Aim2* gene, which activates caspase-1 and IL-1β.

### Dysregulation of Immune Functions in Skin Pathophysiological Contexts

Immunity dysregulations are involved in various skin disorders, such as atopic dermatitis, psoriasis, and alopecia areata.

In atopic dermatitis, deficiency in E-cadherin expression by keratinocytes reduces intercellular junctions, which promotes the secretion of pro-inflammatory cytokines, notably IL-25, IL-33, TSLP, and PGD2, and then induces production of IL-13 and IL-5 by ILC2 (59). IL-13 and IL-4 stimulate activated B cells and T cell proliferation, and their overexpression is associated with allergies (60). Notably, a monoclonal antibody directed against the IL-4 receptor α subunit, blocking IL-4 and IL-13 signaling, has been evaluated in patients with atopic dermatitis, with significant improvement in disease severity (61).

Modified immune properties of keratinocytes have also been associated with the pathophysiology of psoriasis (62). Epidermal cells are renewed every 3 to 5 days in case of psoriasis instead of 28 to 30 days in healthy skin (63). This abnormally accelerated cell renewal rate is due to the premature maturation of keratinocytes, induced by an inflammatory cascade involving dendritic cells, macrophages, and T cells. Autocrine and paracrine secretion of IL-1β by keratinocytes auto-induces insulin-independent growth *via* activation of the p38 MAPK signaling pathway, which alters differentiation and consequently participates in the hyper-proliferative state of the epidermis (64). Knowing that HLA-G and PD-L1 are expressed in psoriatic skin, a possible regulatory link between keratinocyte hyper-proliferation and expression of immune checkpoints is a rational hypothesis, which should be investigated (65, 66).

Another example of skin pathology that involves an immune dysregulation is alopecia areata, which is characterized by hair loss in patch areas, notably but not exclusively in the scalp (67). Hair follicles are normally preserved from immune reactions, a phenomenon called immune privilege. The disruption of this immune privilege has been identified as one of the causes of alopecia areata (68). This pathophysiological process involves an abnormal infiltration of T-cells that causes local inflammation and the destruction of anagen hair follicles (67). Another aspect is the expression of MHC by keratinocytes, which promotes the maintenance of autoreactive T cells directed against hair follicles (69).

## Regulation of Immunity and Tolerance Are Key Points for Implementing Skin Cell and Gene Therapy

The high regenerative potential of adult keratinocyte stem cells underlay the development of skin replacement strategies based on autologous skin-substitute grafting, permanently reconstituting the skin in patients with third-degree burns affecting up to 90% body surface (70). Notably, a clinical trial using keratinocyte stem cells and gene therapy succeeded in regenerating the entire epidermis of a child suffering from epidermolysis bullosa (71). Preservation of functional keratinocyte stem cells during the successive steps of the process is a prerequisite for the long-term graft survival, a point that still requires intensive investigation (72), including of the immune properties of stem cells.

Alternatively, frozen cell banks of allogenic keratinocytes may be constituted for standardized skin substitute production, available immediately on demand. Currently, allogenic keratinocytes are only suited for the bioengineering of temporary cutaneous bio-dressings, as the problem of immune rejection limits any long-term reconstitution. Such temporary dressings are an option for the treatment of chronic venous leg ulcers and diabetic foot ulcers, where the living cells of the allogenic graft stimulate regenerative mechanisms and contribute to restoring the patient’s skin healing functions (73). In addition to native keratinocytes obtained from skin biopsies, keratinocytes generated by differentiation of pluripotent embryonic stem cell (ESC) lines have been investigated as a source of allogenic cells for skin-substitute bioengineering (74).

Transplant rejection is explained by the allelic differences between donor and recipient at the level of the polymorphic loci of three classes of histocompatibility antigen: the ABO blood group, the major histocompatibility complex (MHC) and minor histocompatibility antigens (mHA) (75). In this regard, recognition of allo-HLA antigens by recipient T-cells is the central event initiating allograft rejection. Alloantigen recognition occurs *via* two mechanisms: the direct and indirect allorecognition pathways. Direct recognition consists in T-cell recognition of determinant peptides on intact donor MHC molecules displayed on the surface of the donor antigen-presenting cell (APC), while indirect recognition consists in recognition of determinant allo-peptides presented by the self-MHC on the recipient APC. Secretion of anti-HLA antibodies directed against the HLA donor system leads to graft rejection *via* recruitment of phagocytes or activation of the complement system (76). Experimental approaches have been developed to prevent allogeneic skin graft rejection. Rapamycin, an inhibitor of T-cell proliferation, inhibited rejection in a mouse model (77) in association to IL-2, which controls Treg activity and promotes immune tolerance. There have been a few studies, in a small number of burn patients, using immunosuppressants (methylprednisolone, cyclosporine, prednisone, anti-thymocyte globulin and azathioprine), but this approach is still very limited (78).

## New Strategies to Promote Tolerance for Skin Cell and Gene Therapy Are Required

### Reducing Antigen Presentation

**Figure 2:**
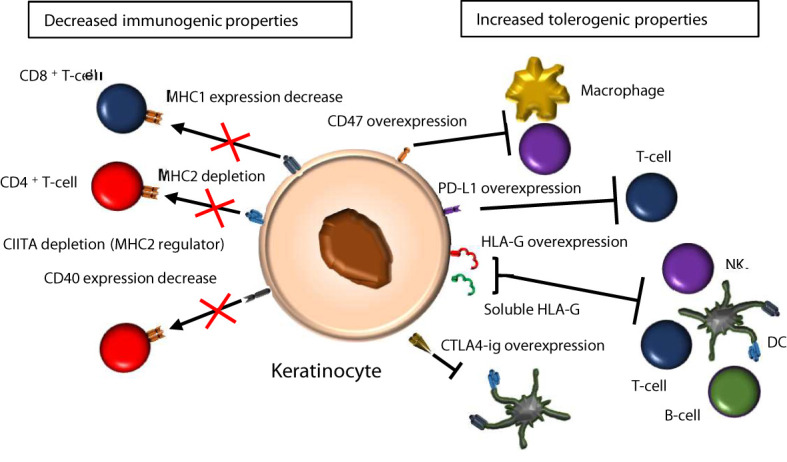
Candidate immunomodulatory strategies in epidermis. There are two main strategies to reduce keratinocyte immunogenicity for epidermis grafting. The first one is to decrease MHC1, MHC2 and co-stimulatory molecules such as CD40, which limits the ability of keratinocytes to present antigens to T-cells. The second one is to overexpress HLA-G and other immune checkpoint molecules (CD47, PD-L1, CTLA4), to increase tolerance.

HLA gene genome-editing has been implemented in induced pluripotent stem cells (iPSCs) to generate universal donor stem cells (79). One strategy consisted in producing pseudo-homozygous cells for the HLA class I genes, from heterozygous donors, by editing the targeted allele. A second approach, taking account of the pivotal role of HLA-C in the suppression of NK cells, consisted in suppressing HLA-A and HLA-B while retaining the HLA-C haplotype, increasing compatibility. In both cases, genome-edited cells were able to suppress T-cell and NK activity, while preserving HLA expression and antigen presentation. This strategy can be combined to MHC2 reduction by depletion of CIITA (**Figure 2**).

Alternatively, iPSC lines overexpressing the immune-suppressive molecule CD47, together with decreased MHC1 and MHC2 expression, have been bioengineered to generate hypo-immunogenic derivatives that attenuate rejection (80). In this strategy, genome-editing comprises overexpression of *CD47* cDNA, which inhibits phagocytosis and NK activity. It is combined with CRISPR directed against B2M to decrease MHC1 expression, and CRISPR directed against the CIITA regulator to decrease MHC2 expression.

### Over-Expressing Immunosuppressive or Immune Checkpoint Molecules

The programmed death-ligand 1 (PD-L1) immune checkpoint molecule is known to bind to the PD-1 receptor expressed at the surface of T-cells, inhibiting their activity (81) and autoimmune reactions (82). PD-L1 promotes tolerance when expressed on the keratinocyte cell surface, by activating IL-10-secreting T-cells (83) and limiting CD4^+^ T-cell proliferation (84).

Bioengineering ESC lines expressing immunosuppressive molecules is an alternative approach for generating universal donor pluripotent stem cell sources. One approach was based on the immunosuppressive properties of CTLA4-Ig, a fusion protein between the extracellular domain of cytotoxic T-lymphocyte antigen 4 (CTLA-4) and an immunoglobulin Fc portion that disrupts T-cell costimulatory pathways, combined to inhibition of T-cell activation *via* PD-L1 (85). High constitutive expression of both CTLA4-Ig and PD-L1 are needed to confer immune protection on hESCs and their derivatives, as these molecules are not sufficient individually.

### Targeting HLA-G Immune Checkpoint Molecules

Human leukocyte antigen-G (HLA-G) molecules are major candidates for implementing immunomodulation strategies since the initial demonstration of the role of HLA-G in protecting the fetus from maternal uterine natural killer cytolysis (86). Application of HLA-G tolerogenic properties was demonstrated in murine models of allogenic tissue transplantation and is also supported by clinical data from transplanted patients. In lung transplant recipients, HLA-G was identified as a predictive marker of low chronic rejection risk (87). In heart transplant recipients, detection of HLA-G expression in sera and endomyocardial biopsies was associated with fewer rejection episodes and suppression of the allogeneic T-cell proliferation response (88). HLA-G was detected in only 20% of samples from patients with heart transplantation, but 86% of patients without acute rejection expressed HLA-G. Similar studies on liver, lung and kidney transplants have also shown a decrease in transplant rejection associated with the presence of HLA-G (89). Thus, low plasma levels HLA-G were proposed as a predictive marker of low risk of acute and chronic kidney rejection (90). Moreover, genomic studies highlighted markers of susceptibility to acute kidney rejection, as specific *HLA-G* gene polymorphisms were shown to participate in the lack of protection against a high risk of transplant rejection (91).

For skin regeneration, several approaches have been developed to use HLA-G as an immunoregulatory agent. A transgenic mouse model expressing HLA-G was designed to investigate immunosuppression in allogenic skin transplantation. In these mice, skin allograft survival was increased in response to HLA-G, which benefit was associated with attenuated T-cell activity (92). Human epithelial cells derived from the umbilical cord, which are known to promote epidermal reconstitution in organotypic models, express HLA-G and HLA-E, lack HLA-DR and several costimulatory molecules, and have a low capacity for presenting antigens. Interestingly, they exerted an inhibitory effect on alloproliferation of PBMCs, which suggested an immunosuppressive function (93). The immune modulatory properties of HLA-G were investigated in a cellular model of human adult skin keratinocytes, in which its expression could be modulated by a doxycycline-inducible construct. When HLA-G expression was induced, keratinocytes exhibited increased inhibition of CD4^+^ T-cell proliferation (84). With the largest aim to generate universal donor pluripotent stem cell sources, human ESCs have been bioengineered to express a mutated form of HLA-G (mHLA-G) exhibiting enhanced mRNA expression and stability, and increased levels of cell-surface HLA-G protein (94). Expression of mHLA-G did not alter the capacity of ESCs to acquire keratinocyte markers in a culture condition directing epithelial orientation. In a mixed lymphocyte reaction assay, ESCs and their keratinocyte derivatives expressing mHLA-G restrained T-cell proliferation and cell lysis driven by allogeneic NK, demonstrating a decreased immunogenicity.

Another proposed approach consisted in using synthetic forms of HLA-G to inhibit transplant rejection. Producing HLA-G as a clinical grade molecule is notably impaired by its limited stability. Thus HLA-G-mediated promotion of immune tolerance was explored using HLA-G-derived synthetic polypeptides as a coating on microbeads suitable for intraperitoneal injection. Mice that received polypeptide‐coated beads acquired tolerance to skin allografts, which resulted in prolonged graft survival (95). Thus, these different approaches point on the HLA-G research field as a promising domain for designing tools aiming at controlling the immunogenicity and the immunosuppressive properties of human keratinocytes.

Skin fibroblasts are also used in skin bioengineering approaches, and are thus concerned by the question of immune tolerance. The generation of fibroblasts expressing a stabilized form of HLA-G has been proposed to reduce their alloreactivity. To engineer a stable HLA-G molecule, mutated HLA-G1 was produced by modifying the endoplasmic reticulum retrieval motif, which allows its increased membrane expression, and the 3 ′UTR region miRNA binding site, which limits regulation by miRNAs. Dermal fibroblasts expressing this modified HLA-G1 were less sensitive to lysis by IL-2-stimulated NKs and reduced the proliferation of PBMCs following activation with PHA (96).

### HLA-G and the Risk of Post-Transplant Cancers

One point to take into account is to prevent the development of post-transplant cancer. Adult keratinocyte stem cells can drift into cancer cells, leading to cutaneous squamous cell carcinoma or basal cell carcinoma development (97, 98). Tumor growth is known to be enhanced by cancer cell ability to escape elimination by the immune system (99). HLA-G and PD-L1 inhibit different populations of T cells in cancer (100, 101), and therefore critically contribute to tumor escape from immunosurveillance. PD-L1 and HLA-G expression and targeting were particularly well documented in squamous cell carcinoma (102) and melanoma (103). It is therefore important to limit the development of post-transplant cancer, as HLA-G and PD-L1 may favor the immune escape of tumor cells. This point was investigated by HLA-G polymorphism matching in heart transplantation, in which recipient and donors were genotyped. Donor-recipient 14 bp polymorphism matching correlated with a limitation of the risk of tumor development post-cardiac transplant (104). It would be interesting to develop a similar approach in case of skin transplantation.

## Concluding Remarks

The role of epidermal cells in skin regeneration is well known; however, their functions in immunity and tolerance mechanisms are under-estimated. Keratinocytes are not merely a structural barrier against environmental insult, but also active members of the sophisticated immune ecosystem in the skin. They actively participate in protective immunity, are involved in the initiation of inflammatory processes, promote long-term maintenance of a memory T-cell pool, and develop their own epigenetic stress memory. As keratinocytes are key players in immune tolerance, they are major targets for overcoming graft rejection in the various strategies that aim to generate bioengineered banks of cells that could be used as universal donor cell sources. In these strategies, modulation of HLA-G expression and function is a promising means of controlling cell immunogenicity, and is being explored in native skin keratinocytes and keratinocytes generated by lineage-oriented differentiation of pluripotent stem cells.

## Author Contributions

All authors contributed to the article and approved the submitted version.

## Funding

This work was supported by CEA funds, including a CFR Ph.D. program grant (2018-2021).

## Conflict of Interest

The authors declare that the research was conducted in the absence of any commercial or financial relationships that could be construed as a potential conflict of interest.

## Publisher’s Note

All claims expressed in this article are solely those of the authors and do not necessarily represent those of their affiliated organizations, or those of the publisher, the editors and the reviewers. Any product that may be evaluated in this article, or claim that may be made by its manufacturer, is not guaranteed or endorsed by the publisher.
